# Combined aromatase, CDK4/6 and PI3K blockade using letrozole/abemaciclib/LY3023414 in endometrial cancer

**DOI:** 10.1016/j.gore.2024.101348

**Published:** 2024-02-22

**Authors:** Panagiotis A. Konstantinopoulos, Niya Xiong, Carolyn Krasner, Joyce F. Liu, Hannah Sawyer, Madeline Polak, Hope Needham, Megan Geddes, Lani Koppermann, Meghan Shea, Cesar Castro, Su-Chun Cheng, Ursula A. Matulonis, Elizabeth K. Lee

**Affiliations:** a Dana-Farber Cancer Institute, Boston, MA, USA; b Beth Israel Deaconess Medical Center, Boston, MA, USA; c Massachusetts General Hospital, Boston, MA, USA

**Keywords:** Abemaciclib, Aromatase inhibition, CDK4/6 inhibition, PI3K inhibition, LY3023414, Endometrial cancer

## Abstract

Several lines of preclinical evidence indicate that combining PI3K and CDK4/6 inhibitors may further enhance the efficacy of hormonal therapy by overcoming *de novo* and acquired resistance to PI3K and CDK4/6 blockade. We evaluated the combination of abemaciclib, letrozole and LY3023414 (an orally available, selective inhibitor of the class I PI3K isoforms and mTORC1/2) in recurrent endometrial cancer (EC). This study was terminated prematurely after 5 patients initiated protocol therapy due to discontinuation of further development of LY3023414. We report our findings from these patients, including one with recurrent endometrioid EC with *AKT1, CTNNB1* and *ESR1* hotspot mutations who had previously progressed through letrozole/everolimus and achieved a partial response to letrozole/abemaciclib/LY3023414.

## Highlights

• We report a patient with recurrent endometrioid EC with *AKT1, CTNNB1* and *ESR1* hotspot mutations.
• This patient had previously progressed after responding to letrozole/everolimus.
• Patient exhibited partial response to combined aromatase, CDK4/6 and PI3K blockade with letrozole/abemaciclib/LY3023414.
• Combining PI3K and CDK4/6 inhibitors may further enhance the efficacy of hormonal therapy.

## 1 Introduction

Approximately 84 % of FIGO grade 1/2 and 50 % of FIGO grade 3 endometrioid endometrial cancers (EC) express estrogen receptor (ER) and are considered to be hormonally driven (Westin and Broaddus, 2012). Various forms of hormonal therapy have been evaluated in EC, including aromatase inhibitors (AI), tamoxifen and progestin as monotherapies as well as letrozole/everolimus and megestrol/tamoxifen combinations (Rose et al., 2000, Slomovitz et al., 2015). However, responses to hormonal/endocrine therapy are typically modest [<10 % objective response rate (ORR) with AI monotherapy], relatively brief in duration and most frequently observed in patients with FIGO grade 1 or 2 tumors (Rose et al., 2000).

We recently reported an investigator-initiated, single arm, phase 2 study in ER positive EC where letrozole/abemaciclib demonstrated an ORR of 30 % and a median PFS of 9.1 months (Konstantinopoulos et al., 2023). Responses were observed regardless of grade, receipt of prior hormonal therapy, or mismatch repair and progesterone receptor (PR) status, thereby providing proof of concept for the mechanistic rationale behind combined aromatase and CDK4/6 inhibition in this setting. However, despite this promising activity, most patients either did not respond or developed resistance to this regimen highlighting the need for novel strategies to overcome *de novo* and acquired resistance.

One such strategy may be the addition of PI3K pathway inhibition to hormonal therapy and CDK4/6 inhibition. First, PI3K pathway alterations are present in ∼ 90 % of endometrioid ECs (Kandoth et al., 2013) and there is extensive crosstalk between the ER and PI3K/AKT/mTOR pathways, i.e., bidirectional signaling between these two pathways whereby PI3K pathway activation is associated with resistance to hormonal therapy, while upregulation of ER signaling by PI3K inhibition mediates resistance to PI3K inhibitor monotherapy (Vasan et al., 2019). Addition of PI3K pathway inhibition has been shown to enhance response to hormonal therapy in breast cancer and the addition of the mTOR inhibitor everolimus to letrozole has demonstrated an ORR of 32 % in recurrent and metastatic EC (Slomovitz et al., 2015). Second, given that PI3K pathway inhibition may be bypassed by activation of the RAS pathway (RTK/RAS/b-catenin pathway alterations are present ∼ 80 % of endometrioid EC) and given that both the PI3K and MAPK pathways converge into overexpression of cyclin D1, a promising approach would be to combine a PI3K inhibitor with a CDK4/6 inhibitor (Du et al., 2020, Alves et al., 2021, Goel et al., 2018). Preclinical studies have demonstrated that CDK4/6 inhibition sensitizes PI3K-altered tumors to PI3K inhibition and, conversely, mTORC1/2 inhibition exhibits anti-tumor activity against CDK4/6 inhibitor resistant cells (Michaloglou et al., 2018). Furthermore, CDK4/6 inhibition has been shown to induce an escape pathway involving PI3K pathway activation, suggesting that PI3K pathway activation may be a mechanism of early adaptive resistance to CDK4/6 blockade (Herrera-Abreu et al., 2016). For these reasons, we postulated that combined PI3K and CDK4/6 inhibition [using LY3023414 (an orally available, potent selective inhibitor of the class I PI3K isoforms, mTORC1/2, and DNA-PK) and abemaciclib respectively] may further augment the efficacy of hormonal therapy compared to PI3K and CDK4/6 blockade alone.

## 2 Methods

Eligible participants had recurrent or metastatic, pathologically confirmed EC, of any histology, that was ER-positive EC (≥1% of tumor cell nuclei being immunoreactive). Other eligibility criteria included measurable disease by RECIST 1.1, no upper limit of prior therapies, ECOG performance status of ≤ 1, and normal organ and marrow function. Previous hormonal therapy, including prior letrozole, was allowed. Key exclusion criteria included prior treatment with any CDK4/6 inhibitor, prior PI3K inhibitor therapy (prior mTOR inhibitors were allowed) and known brain metastases.

The primary objective was objective response by Response Evaluation Criteria in Solid Tumors (RECIST) 1.1 or progression-free survival (PFS) for ≥ 6 months after initiating therapy. Secondary endpoints included PFS, overall survival (OS), and toxicity.

Participants received abemaciclib at 150 mg po twice daily (BID), LY3023414 100 mg po BID and letrozole at 2.5 mg po once daily (dose level 0/DL0) with a safety lead-in following a standard 3 + 3 trial design. If DL0 was tolerated, LY3023414 would be escalated to 150 mg BID (DL1); otherwise abemaciclib would be de-escalated to 100 mg BID (DL-1).

A small exploratory cohort of participants with ER-negative disease but with genomic PI3K pathway alterations was also included; these patients received abemaciclib and LY3023414, without letrozole.

This investigator-initiated study (IND holder PK) was open at 3 institutions (Beth Israel Deaconess Medical Center, Dana-Farber Cancer Institute and Massachusetts General Hospital) and was approved by the institutional review board (IRB) of Dana-Farber Harvard Cancer Center (NCT03675893). Written informed consent was obtained from all patients or their guardians before enrollment in the study. The study was funded by Eli Lilly and Company (Lilly), which also provided abemaciclib and LY3023414. Participants were provided with commercially available letrozole.

## 3 Results

Before clinical development of LY3023414 was terminated, 4 patients (all with endometrioid ECs) were enrolled and initiated therapy in the ER + cohort and 1 patient (with serous EC) in the ER- cohort, Table 1.

**Table 1:** Clinical, Pathologic and Response Data of Enrolled Patients.

Patient	Cohort	Age	ECOG	Histology	Prior lines	Best response^b^	Off Treatment Reason	Survival status	PFS (months)	OS (months)
1	ER+	62.3	1	Endometrioid G3	2	N/E^a^	Withdrew from Study	Withdrew Consent for Follow-Up	N/E^c^	0.62
2	ER+	66.4	1	Endometrioid G2	6	N/E^a^	Clinical Progressive Disease	Dead	N/E^d^	2.79
3	ER+	59.8	0	Endometrioid G1	2	Stable Disease	Withdrew from Study	Alive	1.94	37.16+
4	ER-	67.4	1	Serous	2	Progressive Disease	Progression per RECIST 1.1	Dead	0.3	0.76
5	ER+	63.8	0	Endometrioid G1	4	Partial Response	Progression per RECIST 1.1	Alive	10.84	35.06‡

a N/E = non-evaluable.

b By RECIST 1.1.

c Patient 1 withdrew consent 16 days after initiating therapy because of personal decision.

d Patient 2 came off study 28 days after initiating therapy because of clinical progression.

† As of the last follow-up date of 03–01-2022.

‡ As of the last follow-date of 01–28-2022.

All 5 patients eventually discontinued protocol therapy, 2 because of disease progression by RECIST 1.1, 1 because of clinical progression and 2 because of withdrawal of consent (one because of logistical reasons and one because of fatigue). Three patients had restaging scans during the protocol therapy (1 patient withdrew consent and 1 had clinical progression before reaching first scan); 1 patient had partial response (PR, ER + cohort), 1 patient had stable disease (ER + cohort), and 1 patient had progressive disease (ER- cohort) as best response, Table 1. The patient with PR had previously progressed through letrozole/everolimus and had a grade 1 endometrioid tumor with hotspot *AKT1* E17K, *ESR1* Y537S and *CTNNB1* S33F mutations. Her tumor was mismatch repair proficient, and *POLE* and *TP53* wildtype, corresponding to the no-specific-molecular profile (NSMP) EC group. This patient developed radiographic progression after 10.84 months.

Treatment-related adverse event (TRAE) that were at least Grade 2 are presented in Table 2. There were no Grade 4 or 5 toxicities. Grade 3 TRAEs included fatigue (n = 3 patients), decreased platelet count (n = 1) and hypokalemia (n = 1). Diarrhea, a known toxicity of abemaciclib and LY3023414 was observed in 2 patients (1 G1 and 1 G2). Given that abemaciclib inhibits renal transporters OCT2, MATE1, and MATE2-K, increases in creatinine (1 G1 and 2 G2) were observed in 3 patients; these patients were monitored, and creatinine increases were reversible upon holding and/or discontinuation of abemaciclib dosing.

**Table 2:** Treatment-Related Adverse Events (TRAEs) of at least Grade 2 in any patient (by maximum grade).^a^

**Adverse Events**	**Grade 1**	**Grade 2**	**Grade 3**
Fatigue	0	0	3
Platelet count decreased	0	0	1
Hypokalemia	0	0	1
Anemia	1	2	0
Creatinine increased	1	2	0
Diarrhea	1	1	0
Nausea	1	1	0
Dehydration	1	1	0
Hyperkalemia	1	1	0
Colitis	0	1	0
Dyspepsia	0	1	0
Neutrophil count decreased	0	1	0
Hyponatremia	0	1	0
Hematuria	0	1	0

a There were no G4 or G5 adverse events.

## 4 Discussion

Preclinical evidence suggest that combining PI3K and CDK4/6 inhibitors may enhance the activity of hormonal therapy by overcoming *de novo* and acquired resistance to PI3K and CDK4/6 blockade monotherapy. Unfortunately, this study of letrozole/abemaciclib/LY3023414 was terminated prematurely due to discontinuation of further development of LY3023414, thereby precluding any conclusions regarding the efficacy and safety of this regimen in EC. However, one patient achieved a partial response to letrozole/abemaciclib/LY3023414 and remained on protocol therapy for more than 10 months. This patient had previously participated in a clinical trial of the dual mTOR inhibitor MLN0128 to which she had an excellent response but discontinued protocol therapy due to intolerable diarrhea and fatigue. She subsequently switched therapy to letrozole/everolimus to which she remained for more than a year before developing progressive disease.

Review of this patient’s targeted next generation sequencing results yielded several interesting observations. Specifically, her tumor harbored an *AKT1* point alteration in the pleckstrin homology domain (E17K) and leads to constitutively active PI3K signaling by promoting localization of AKT to the plasma membrane in a PI3K-independent manner. This is the most common *AKT1* alteration across tumors, observed in approximately 2–7 % of breast cancers (exclusively in hormone positive tumors) and in up to 4 % of endometrial cancers (4 % in the MSK IMPACT and 0 % in TCGA) (Gao et al., 2013). Interestingly, the presence of this hotspot *AKT1* E17K mutation has been associated with clinical advantage with mTOR inhibition in isolated case reports and in the GENIE clinicogenomics registry (Smyth et al., 2020), an observation that may explain the excellent response of this patient initially to the dual mTOR inhibitor MLN0128 and subsequently to everolimus.

Additionally, this patient’s tumor harbored the hotspot *CTNNB1* S33F alteration, a gain-of-function mutation that prevents phosphorylation of beta-catenin by glycogen synthase kinase-3 (GSK3) at the S33 phosphorylation site which is critical for the detection of the beta-catenin protein by the E3-ubiquitin ligase β-TrCP (Razak et al., 2019). *CTNNB1* is one of the most frequently mutated genes in EC, especially in the NSMP molecular subgroup where it is mutated in the 52 % of the tumors included in TCGA (Kandoth et al., 2013, Gao et al., 2013). Importantly, in a previous study, patients with *CTNNB1*-mutated endometrioid EC responded well to everolimus/letrozole (Slomovitz et al., 2015); this may reflect the fact that accumulated beta-catenin has been reported to also result in mTORC1 activation and thus response to mTOR inhibition (Fujishita et al., 2008). Therefore, presence of the *CTNNB1* mutation may explain the excellent response of this patient initially to MLN0128 and then to everolimus.

Eventually, this patient developed resistance to letrozole/everolimus that was overcome by letrozole/abemaciclib/LY3023414. Key mechanisms of resistance to everolimus include the development of *mTORC1* mutations as well as feedback activation of the PI3K signaling pathway and paradoxical activation of MAPK/ERK signaling. Of these resistance mechanisms, feedback activation of PI3K signaling is the most common, and may have been circumvented by the dual PI3K/mTOR inhibitor LY3023414 which targets class I PI3K isoforms and mTORC1/2. Unlike the allosteric mTOR inhibitor everolimus which predominantly targets mTORC1, LY3023414 targets the ATP binding site of mTOR resulting in significant decrease in both mTORC1 and mTORC2 kinase activity, which, together with inhibition of class I PI3K isoforms, facilitates more effective and comprehensive targeting of the PI3K signaling pathway that may have overcome the feedback loops that conferred resistance to everolimus in this patient.

Alternatively, resistance to everolimus in this patient may have been overcome by abemaciclib, given that paradoxical activation of both PI3K and MAPK pathways converges into overexpression of cyclin D1, which in turn activates CDK4, phosphorylates RB and permits initiation of the cell cycle, an event that can be overcome by CDK4/6 blockade. This is particularly relevant in the setting of the *CTNNB1* S33F mutation (present in this patient’s tumor) because cyclin D1 is a key target gene of the TCF/LEF-1 transcription factor that is activated in the setting of beta-catenin accumulation caused by the *CTNNB1* S33F mutation as described above. Of note, in our previous letrozole/abemaciclib study, all *CTNNB1*-mutated tumors exhibited a partial response to that regimen (Konstantinopoulos et al., 2023). Unfortunately, no baseline biopsy prior to initiation of letrozole/abemaciclib/LY3023414 was available for this patient (or any of the patients in the study), so no functional studies to determine the exact mechanism of acquired resistance to letrozole/everolimus could be performed. Therefore, it is impossible to tell which component of the letrozole/abemaciclib/LY3023414 regimen, was responsible for overcoming resistance to everolimus in this patient.

Finally, another clinically important finding was the presence of the *ESR1* Y537S mutation. This is a well characterized (structurally and functionally) alteration in the ligand binding domain (LBD) of ER which maintains ER in a permanently agonist conformation leading to ligand-independent activity that is resistant to aromatase inhibitor (AI) therapy (Robinson et al., 2013, Stover et al., 2018, 2018.). In breast cancer, *ESR1* mutations are relatively uncommon in primary, untreated tumors (∼1–3 % of primary breast cancers) but are prevalent in metastatic breast tumors (∼20 %), especially those that have been exposed to AI therapy. Interestingly, the *ESR1* Y537S mutation was identified in this patient’s tumor specimen from the initial diagnosis, before any exposure to hormonal therapy; only 6 % of primary NSMP ECs exhibit *ESR1* LBD mutations in TCGA (Kandoth et al., 2013, Gao et al., 2013). Mechanistically, by targeting cyclin D1, which is a key downstream ER target gene, abemaciclib may overcome ligand-independent activation of the ER transcriptional activity (conferred by *ESR1* mutations), and studies in breast cancer have demonstrated that the benefit of addition of CDK4/6 blockade to hormonal therapy is observed irrespective of *ESR1* mutation status (Tolaney et al., 2022). However, ESR1 mutations confer resistance to AIs, so drugs that directly target the ER such as the selective ER degraders (SERDs) fulvestrant and elacestrant are preferable in this setting. Elacestrant has been shown to be superior to fulvestrant and has received regulatory approval in the United States for the treatment of *ESR1* mutated breast cancer (Bidard et al., 2022). Therefore, after progression through protocol therapy with letrozole/abemaciclib/LY3023414, incorporation of elacestrant (or another SERD) as the backbone of any future hormonal therapy combination may be a good option for this patient. Accordingly, the PADA1 trial demonstrated that development of an ESR1 mutation while on AI plus CDK4/6 inhibitor therapy may be overcome by switch from AI to fulvestrant in combination with the same CDK4/6 inhibitor (Bidard et al., 2022).

Moving forward, there remains a significant interest in combinations of CDK4/6 and PI3K inhibitors to augment hormonal therapy. We are currently conducting a letrozole/abemaciclib/metformin in ER + endometrioid EC which is expected to report next year. Furthermore, mutant- and isoform-selective PI3K inhibitors with a much more favorable safety profile compared to earlier PI3K inhibitors, renders them ideal partners for future combinations with CDK4/6 inhibitors.

## 5 Consent

Written informed consent was obtained from all patients or their guardians before enrollment in the study.

## Author contributions

All authors contributed to the concept, data collection, interpretation of data and writing of the manuscript. All authors approved the final version of the manuscript.

## Declaration of competing interests

Niya Xiong, Carolyn Krasner, Hannah Sawyer, Madeline Polak, Hope Needham, Megan Geddes, Lani Koppermann and Su-Chun Cheng have nothing to disclose.

Megan Shea reports participation on a Data Safety Monitoring Board or Advisory Board from GSK and Eisai, and support for attending meetings and/or travel from Massachusetts Society of Clinical Oncology.

Joyce Liu reports funding to Dana-Farber Cancer Institute for Trials on which she is the PI from 2X Oncology, Aravive, Arch Oncology, AstraZeneca, Bristol-Myers Squibb, Clovis Oncology, GlaxoSmithKline, Impact Therapeutics, Regeneron, Seagen, Vigeo Therapeutics, and Zentalis Pharmaceuticals; she also reports consulting and/or advisory board participation from AstraZeneca, Bristol-Myers Squibb, Clovis Oncology, Daiichi Sankyo, Eisai, Genentech, GlaxoSmithKline, Regeneron Therapeutics and Zentalis Pharmaceuticals; she also reports being Co-chair of the NRG Oncology Ovarian Subcommittee and Co-chair of Tina’s Wish Scientific Advisory Board.

Panagiotis Konstantinopoulos reports funding to Dana-Farber Cancer Institute from AstraZeneca, Pfizer, Eli Lilly, Bayer, Merck, GSK, Tesaro, Merck KGaA on trials which he is the PI; he also reports consulting and/or advisory board participation from Immunogen, GSK, Novartis, Alkermes, AstraZeneca, Bayer, Merck, Pfizer, Tesaro, Vertex, EMD Serono, Kadmon, BMS, IMV, Repare, Artios, Mersana.

Ursula Matulonis reports consulting fees from Merck, GSK, AstraZeneca, Pfizer; payment on a CME lecture and slides on endometrial cancer from Med Learning Group; travel support from Immunogen to travel to an FDA launch meeting; she also reports fees from participation in advisory boards from Allarity, NextCure, Trillium, Agenus, Profound Bio, Novartis, Boerhinger Ingelheim, Rivkin Foundation, Ovarian Cancer Research Alliance, Clearity Foundation, Morphosy, CureLab, Eisai and fees from participation in Data Safety Monitoring Boards from Alkermes and Symphogen.

Cesar Castro reports consulting fees from Qiagen, Teladoc and Advanced Medical.

Elizabeth Lee reports funding to Dana-Farber Cancer Institute for a clinical Trial on which she is the PI from Merck; she also reports consulting fees from Aadi Biosciences and a GOG New Investigator Award for travel support to attend NRG Oncology conferences.

## CRediT authorship contribution statement

**Panagiotis A. Konstantinopoulos:** Conceptualization, Data curation, Formal analysis, Investigation, Supervision, Writing – original draft, Writing – review & editing. **Niya Xiong:** Data curation, Formal analysis, Writing – review & editing. **Carolyn Krasner:** Data curation, Writing – review & editing. **Joyce F. Liu:** Data curation, Writing – review & editing. **Hannah Sawyer:** Data curation, Formal analysis, Writing – review & editing. **Madeline Polak:** Data curation, Formal analysis, Writing – review & editing. **Hope Needham:** Data curation, Formal analysis, Writing – review & editing. **Megan Geddes:** Data curation, Formal analysis, Writing – review & editing. **Lani Koppermann:** Data curation, Formal analysis, Writing – review & editing. **Meghan Shea:** Data curation, Formal analysis, Writing – review & editing. **Cesar Castro:** Data curation, Writing – review & editing. **Su-Chun Cheng:** Data curation, Formal analysis, Writing – review & editing. **Ursula A. Matulonis:** Data curation, Formal analysis, Funding acquisition, Writing – review & editing. **Elizabeth K. Lee:** Conceptualization, Data curation, Formal analysis, Writing – review & editing.

## Declaration of competing interest

The authors declare that they have no known competing financial interests or personal relationships that could have appeared to influence the work reported in this paper.
